# Unequal impact and spatial aggregation distort COVID-19 growth rates

**DOI:** 10.1098/rsta.2021.0122

**Published:** 2022-01-10

**Authors:** Keith Burghardt, Siyi Guo, Kristina Lerman

**Affiliations:** ^1^ Information Sciences Institute, 4676 Admiralty Road, Marina del Rey, CA 90292, USA; ^2^ Department of Computer Science, University of Southern California, 941 Bloom Walk, Los Angeles, CA 90089, USA

**Keywords:** COVID-19, aggregation bias, Reed–Hughes mechanism

## Abstract

The COVID-19 pandemic has posed unprecedented challenges to public health world-wide. To make decisions about mitigation strategies and to understand the disease dynamics, policy makers and epidemiologists must know how the disease is spreading in their communities. Here we analyse confirmed infections and deaths over multiple geographic scales to show that COVID-19’s impact is highly unequal: many regions have nearly zero infections, while others are hot spots. We attribute the effect to a Reed–Hughes-like mechanism in which the disease arrives to regions at different times and grows exponentially at different rates. Faster growing regions correspond to hot spots that dominate spatially aggregated statistics, thereby skewing growth rates at larger spatial scales. Finally, we use these analyses to show that, across multiple spatial scales, the growth rate of COVID-19 has slowed down with each surge. These results demonstrate a trade-off when estimating growth rates: while spatial aggregation lowers noise, it can increase bias. Public policy and epidemic modelling should be aware of, and aim to address, this distortion.

This article is part of the theme issue ‘Data science approaches to infectious disease surveillance’.

## 1. Introduction

The COVID-19 pandemic has spread rapidly around the globe, claiming millions of lives and wreaking havoc on world economies. Public health experts and policy makers must consider how quickly a disease is spreading in their communities when deciding what mitigation strategies to implement at what time, such as closing schools or ordering residents to shelter at home.

Epidemiologists and public health experts measure the infection growth rates [1] to better estimate the basic reproduction number of a disease ($R_{0}$) [2] or forecast disease spread [3]. Although growth rates lack important information that other statistics can offer, they offer the advantage of being less susceptible to over-fitting.

We show that the impact of COVID-19 is highly unequal, with *hot spots* (regions with many COVID-19 cases or deaths) emerging at multiple spatial scales [4]: from individual facilities [5] and city neighbourhoods [6], to US counties and states [7], to nations [8]. We also show that spatial aggregation of COVID-19 data across regions leads to higher estimates of growth rates than within most regions—a phenomenon we call *aggregation bias*. As a result of aggregation bias, the growth rates of infections and deaths at the state level are higher than for most counties within each state, and the growth rate at the city level overestimates how quickly the disease spreads through most city neighbourhoods. This is further confirmed through the analysis of three major COVID-19 surges in the USA.

We argue that hot spots and aggregation bias arise because of the heterogeneous growth rate of the disease. Namely, regions where the disease is spreading more quickly grow to become hot spots and dominate statistics. Spatial aggregation of data produces growth estimates that are systematically higher relative to those within most regions due to these hot spots. This offers an explanation for how early epidemics may overestimate the effective reproduction number [9], and demonstrates the trade-off epidemiologists have to make between disaggregation (lower bias) and aggregation (lower noise).

To better understand the emergence of hot spots, we create a simple stochastic model, a variant of the Reed–Hughes mechanism [10], in which the disease arrives at different times in regions and grows exponentially at different rates. The varying ages of outbreaks create a heavy-tailed distribution in the number of infections and deaths, with a small number of hot spots representing the majority of all cases. Combining cases data from simulated regions with varying growth rates can systematically amplify the aggregate growth rates. This is because high-growth regions have more total infections, and therefore dominate the statistics. In addition, we explore these effects for multiple waves in the USA. We find that the growth rates consistently decrease with each additional wave, even as deaths become more prevalent across the nation. That said, we find aggregation bias is preserved for all waves.

Epidemic modelling and public health policy-makers need to account for the role of aggregation bias in the analysis of disease data. When calculating the costs and benefits of lock-downs, for example, analysts must control for these biases to better understand the risks most people face. Aggregating data could also affect parameters in epidemic models and therefore reduce model prediction accuracy.

## 2. Results

**Figure 1.**
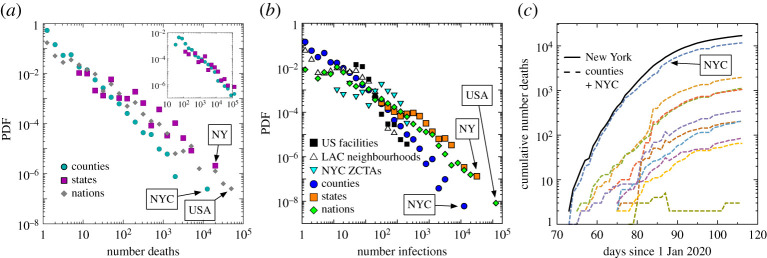
The unequal impact of COVID-19. (*a*) The number of deaths (as of April 2020) has a heavy-tailed distribution for counties, states and nations, with the most cases in New York City, New York State and the USA, respectively. Inset: a stochastic model discussed in the main text qualitatively captures the properties of the distribution. (*b*) A similar pattern is seen for infections at many spatial scales: from US facilities to neighbourhoods to nations. (*c*) Deaths over time for New York state and select counties, where we see the disease initially grows at an approximately exponential rate and the disease arrives in counties at different times. (Online version in colour.)

Figure 1 demonstrates the unequal impact of COVID-19 in the USA and the world in April 2020. The number of deaths in US counties, US states and world nations has a heavy-tailed distribution (figure 1*a*): the disease’s toll varies enormously, with many communities almost unaffected and others hit hard by the pandemic. For example, New York City accounts for the bulk of all deaths in New York state, which accounts for a large fraction of US deaths. The number of infections has a heavy-tailed distribution across *multiple spatial scales* (figure 1*b*): from large outbreaks at US facilities (e.g. nursing homes, prisons), to Los Angeles County (LAC) and New York City (NYC) neighbourhoods, US counties and states, and nations. Despite differences in testing, there exist strong regularities in the prevalence of hot spots at these vastly disparate scales.

Figure 1*c* shows the growing toll of the disease in New York state and its hardest-hit counties through late April 2020. The daily growth of new cases in the outbreak’s early stages can be roughly modelled as an exponential, which allows us to estimate the average growth rate using negative binomial regression (see Methods and materials). The Reed–Hughes mechanism [10] explains how exponentially growing populations of different age produce a power-law distribution of population sizes. We see strong similarities between this mechanism and what we empirically observe: infections and deaths follow a heavy-tailed distribution, grow exponentially and appear in regions at different times. However, we must modify the model to account for the outbreak appearing at unequally distributed times and growth rates varying in each region. Owing to the differences between the real growth behaviour and the Reed–Hughes mechanism, the agreement with a power-law is only qualitative. To validate the modified mechanism, we create synthetic outbreaks, which follow the growth rate calculated for counties. We also aggregate these simulated counties to the state level. In both cases, simulations create a heavy-tailed distribution (figure 1*a* inset), similar to what we see in data.

The deviations from the traditional Reed–Hughes mechanism, especially the variance in growth rates, lead to aggregation bias. Here we calculate the aggregated growth rate of a region (e.g. a state with many counties) by fitting the exponential growth curve of the total number of confirmed infections or deaths in this region. We found that hot spots dominate spatially aggregated data and are correlated with faster growing infections: Spearman’s rank correlation coefficient, $r_{s}$, between infection growth rate and LA County, county, state and nation infections is 0.25–0.6 with p-values<0.0002 in April 2020. Similarly, the correlation between death growth rate and county, state, and nation deaths is 0.51–0.53 with p-values<0.0002. This agrees with the simulation in figure 1*a* inset ($r_{s}=0.90$, $p\text{-value}<10^{-6}$), further demonstrating that this mechanism captures empirical behaviour. The hot spots make spatially aggregated deaths and infections appear to grow faster at the larger scale than disaggregated data.

**Figure 2.**
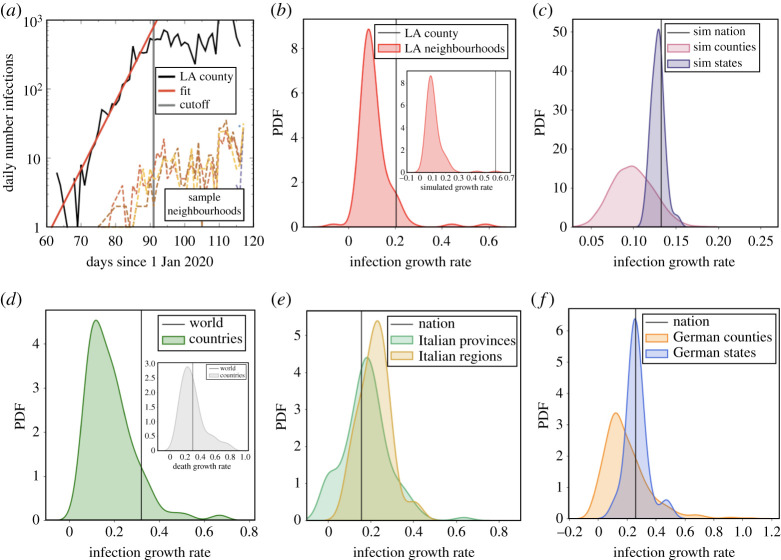
Growth of COVID-19 at different spatial scales. (*a*) We find reasonable agreement between the growth rate of infections (noisy black line) and an exponential growth model (straight fitted line) before the cutoff (vertical grey line). Daily infection rates for a sample of LA county neighbourhoods is also shown. (*b*) The growth rate of individual neighbourhoods and LA county (vertical line), among 116 neighbourhoods with significant cases (see Methods and materials). Inset: we simulate cases in LA neighbourhoods as exponentially growing without statistical noise and find the aggregation bias is preserved. (*c*) The growth rate for counties, states and nation simulated using SIR models, of which the transition rates are drawn from normal distributions. (*d*) The infection growth rate for nations and the world (vertical line). Inset: a plot of the death growth rate for nations and the world. (*e*) The infection growth rate for Italian provinces, regions and the entire Italian nation. (*f* ) The infection growth rate for German counties, states and the entire Germany nation. (Online version in colour.)

The impact of growth rate variance can be seen over a variety of spatial scales in figure 2. Data are fit to the exponential growth phase, as shown in figure 2*a*, for individual neighbourhoods within Los Angeles (LA) county and for all cases in the county as a whole. The disease growth rate at the neighbourhood level is lower than LA County as a whole during its initial exponential growth period, between March and April 2020 (figure 2*a*,*b*). Figure 2*b* inset shows the distribution of simulated growth in LA County neighbourhoods (arrival times and exponential growth rates are fit to each neighbourhood). In these idealized simulations, infections grow exponentially (without a cut-off) and without noise, which provides some quantitative differences with observational data, but we find qualitatively similar behaviour, including aggregation bias. Namely, for both simulations and empirical LA data, the difference between the aggregated growth rate and the mean of neighbourhood growth rates is statistically significant (difference in mean $p\text{-value}<10^{-10}$; see Methods and materials for calculations of p-values). However, if we only vary arrival times of the disease in different neighbourhoods, but keep the growth rates the same, we instead find that aggregation has a smaller, although statistically significant, impact (see appendix A). These findings suggest variance in growth rate is the primary reason for aggregation bias. We also simulated disease growth using SIR models, and aggregation bias is similarly observed (figure 2*c*). The difference between the distributions at the simulated county and state level are significant (Mann–Whitney U-test p-value $<10^{-10}$). Similarly, the difference between the distribution at the state level and the nation level growth rate is also statistically significant (difference in mean p-value=0.01). The details about the SIR simulations can be found in the Methods and materials section. The systematic overestimation (or underestimation) of growth rates due to spatial aggregation is an example of the Modifiable Areal Unit Problem [11], a statistical bias similar to Simpson’s paradox [12], that results in varying statistical trends at different levels of data aggregation. Overall, these results highlight the dangers of making conclusions from aggregated data.

At a larger scale, growth within nations is lower than the growth within the world as a whole during January to April 2020, when most of nations experienced their first exponential growth in COVID-19 cases (figure 2*d*). The difference between the distribution of country growth rates and the aggregated world-wide growth rate is significant (difference in mean $p\text{-value}<10^{-10}$), although the difference is not statistically significant for deaths, shown in the inset of figure 2*d* (difference in mean p-value=0.98). Figure 2*e*, however, shows that aggregation does not always positively bias the growth rate. In Italy, aggregation at the region level (larger spatial areas) is higher than at the province level (smaller areas), as expected. However, the growth rate at the nation level is *lower* than at the region level. The p-value between growth rates distributions of Italian provinces and regions is significant (Mann–Whitney U-test =0.02), as is the difference between distribution of province growth and the nation-level growth (difference in mean p-value<0.001). The reason for this unusual behaviour is not fully understood, and motivates further study, but still shows how aggregate statistics are not necessarily representative of disaggregated statistics. Finally, our results are confirmed at the county, state and national level in Germany (figure 2*f*), where most counties have growth rates that are lower than within most states (Mann–Whitney U-test p-value $=7\times 10^{-4}$) and lower than the nationally aggregated growth rate, although aggregation at the nation level is close to that at the state level (difference in mean p-value=0.75).

**Figure 3.**
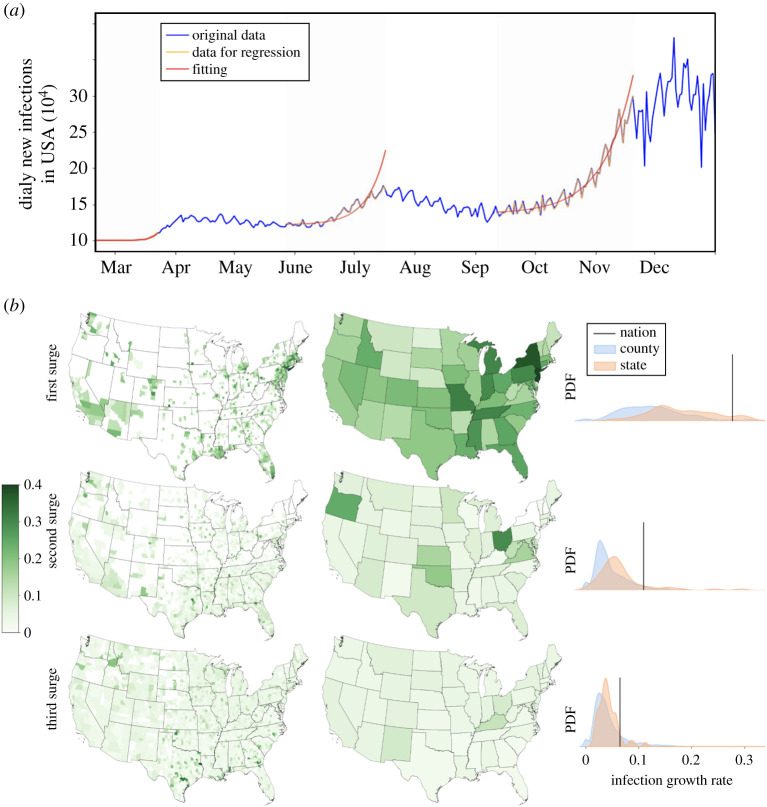
Growth of COVID-19 in the USA. (*a*) The number of daily new infections in the USA showing three surges of exponential growth. We determine the range of the exponential growth period in the data (shaded region) by selecting the best negative binomial fitting (smooth fitted line). (*b*) The maps and distributions of growth rate for county, state and US infections during three surges. (Online version in colour.)

These findings are tested at different stages of the pandemic in figure 3. We fit data in the exponentially growing regime, as illustrated in figure 3*a*. Details about the estimation of growth rates, and how we determine where the disease stops growing exponentially, are discussed in the Methods and materials section. Figure 3*a* shows that USA has experienced multiple exponential surges of COVID-19. This could be explained by government interventions that are dynamically changing over time or seasonal changes in behaviour (e.g. holiday travel). Within the USA, we found that aggregation bias occurred at different spatial scales and during different surges (figure 3*b*). During all three surges, disease growth rate in counties is lower than in states (Mann–Whitney $U\;\text{test}\;p\text{-value}<10^{-4}$ except for third surge county and state growth rates where the p-value=0.49), which is in turn lower than in the entire country (difference in mean p-value is $<10^{-4}$). Deaths data in counties and states shows agreement with our findings for infections (see appendix A). We also find that the growth rates are largest in the first surge and decrease in later surges for counties, states, and the entire USA. There is a significant difference between growth rate distributions in the first and second surge and between the second and third surge for counties as well as states (Mann–Whitney U-test p-value $<10^{-4}$). At the national level, the difference in calculated growth rate exponents between the first and second surges as well as between the second and third surges is also significant (difference in mean $p\text{-values}<10^{-10}$). We also notice in figure 3*b* that variance in the growth rate appears to decrease in later surges. One explanation for these findings is that people are more conscious about the disease as time passes and are therefore more likely to change their behaviour, such as wear a mask and physically distance from others, which lowers the growth rates, and as more people follow this convention, the growth rates become more uniform. Another possible explanation for the lower growth rates is that people who are most likely to become infected (e.g. hubs in the contact network) were infected early on [13,14], while the less susceptible individuals were infected later. Previous work finds that if simulated networks have fewer hubs (and lower second degree moment) the growth rate is lower [14], thus potentially explaining why subsequent surges have a lower growth rate. Despite lower growth rates in later surges, the case counts are often much higher (figure 3*a*), because each surge starts at a higher number of daily cases before the growth begins, highlighting the importance of reducing the infection base rate when controlling the outbreaks.

The aggregation bias and the decrease of growth rates over time can be further demonstrated in figure 3*b*. Comparing the state and county maps of same surges, especially the first surge, we notice that within a state with high growth rate, often there are only a few hot spots (counties) with high growth, whereas the majority of other counties have moderate to low disease growth. The New York state is a prototypical example. The map also shows that growth rates decrease during later surges across the USA, especially at the state level (second column of figure 3*b*). However, the number of counties with COVID-19 cases increases over time, as shown by the first column of figure 3*b*.

**Figure 6.**
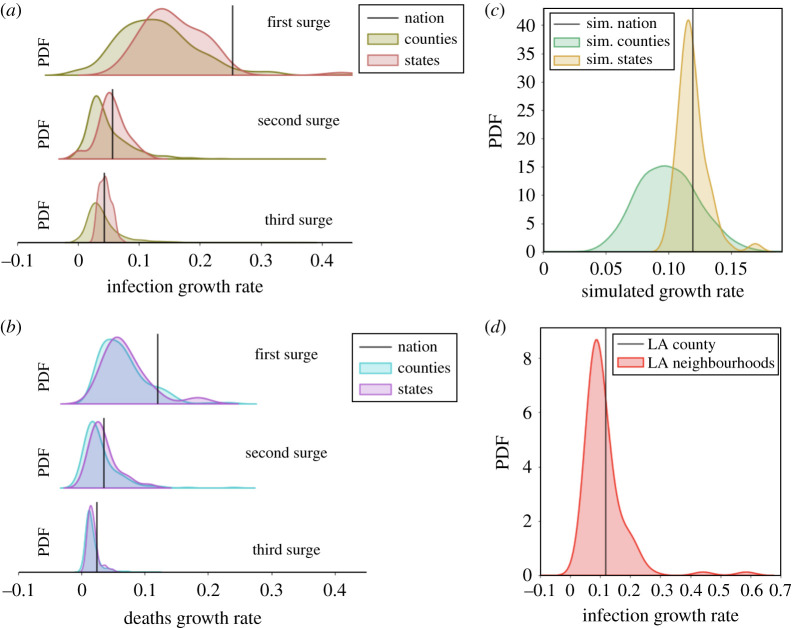
Growth rates with alternative aggregation method. The distributions of (*a*) infections and (*b*) deaths growth rate during three surges in USA, (*c*) the distributions of simulated SIR models for county, state and US nation level, and (*d*) the distributions of infections growth rates in Los Angeles neighbourhoods. The county-level growth rates are estimated by fitting exponential infection curves. The state-level growth rates are estimated by taking the mean of county-level growth rates, weighted by the number of cumulative infections at the end of exponential growth period in each county. The nation-level growth rates are aggregated from state-level growth rates, with the same method. (Online version in colour.)

So far, we have defined the aggregate growth rate of a region to be the exponential growth rate of the daily aggregated number of infections or deaths in a region. This is in contrast to the unweighted mean of growth rates within sub-regions. We can alternatively take the mean of growth rates within sub-regions weighted by the size of the outbreak, i.e. the number of infections (or deaths) at the end of their respective exponential growth periods. We applied this estimation scheme to the empirical data for three COVID-19 surges in the USA and to SIR simulations. We found aggregation bias still exists (figure 6), which demonstrates the robustness of spatial aggregation bias.

Finally, we discuss why growth rates vary. We find that the growth rates within the USA are correlated with population density. The Spearman rank correlation is $r_{s}=0.29$ for counties and 0.49 for states in the first surge (p-values<0.0003), 0.19 for counties ($p\text{-value}<10^{-10}$) and not significant (p-value=0.71) for states in the second surge, and 0.07 (p-value=0.0004) for counties and not significant (p-value=0.35) for states in the third surge. In LA county, this is counter-intuitively negative (−0.19, p-value=0.08, borderline), but is positive (0.09, p-value=0.07, borderline) for counties in Germany. That said, the correlations are not significant for German states, Italian provinces or regions, or, at the world level, nations (p-value>0.45). We see similar results for deaths data (see appendix A). COVID-19 therefore tends to spread more quickly in more densely populated counties and states, in agreement with trends for cities reported by Stier *et al.* [15].

We also find that the arrival date impacts the growth of COVID-19. Namely, communities where the disease arrived earlier tend to have higher growth rates, represented as a negative correlation between the date and the growth rate. For LA neighbourhoods, the correlation between arrival date and growth rate is not statistically significant, but the correlation is −0.31 ($p\text{-value}<10^{-10}$) for counties and −0.25 (p-value borderline, 0.07) for states in the USA. In Germany, the arrival time at the county level is statistically significant (−0.34, $p\text{-value}<10^{-10}$), but this is not statistically significant for the state level, nor is the correlation significant for provinces and regions within Italy. At the world level, however, the correlation is again significant (−0.27, $p\text{-value}=4\times 10^{-4}$). The reason for these correlations is possibly because COVID-19 was spreading before effective mitigation measures were introduced.

## 3. Discussion

COVID-19’s toll around the world varies widely, with many regions initially seeing little impact, while a handful were greatly affected. The heavy-tailed distribution of impact has important implications for decision makers. First, local hot spots, where the virus is far more prevalent, typically grow rapidly. These hot spots bias aggregated growth rates, making COVID-19 appear to grow faster at a larger scale than it does within the constituent communities. The reason for growth rate variation appears to be due to heterogeneity in population size and density, and arrival time of the disease.

Our analysis focuses on data aggregated at different spatial scales. Alternatively, one could explore the growth of spatially distributed transmission trees. While data on large infection trees is unavailable at this time, it will be important future work. The qualitative agreement between empirical data and simulations, which assumes multiple independently growing infections, however, suggests that our results will be robust to this new analysis. Similarly, imported cases have been found to bias aggregate effective reproduction numbers [16]. While we do not in general know what cases were imported, we measure growth rates in regions with at least 30 total infections or deaths, as shown in figures 1*c* and 2*a* (see Methods and materials). Imported cases in these regions are less significant than community spread, therefore our results should be robust to such effects. Moreover, agreement of results in later surges, where imported cases are a smaller fraction of the entire dataset, suggests this is not an important factor. Nonetheless, future work should explore the effect imported cases may have on aggregation biases.

Analysis of the costs and benefits of mitigation strategies, including lock-downs, has to account for potential biases introduced by data aggregation. Local hot spots may amplify estimated disease growth rates, obscuring the benefits of early interventions. Epidemiologists similarly need an accurate understanding of disease growth rates to infer disease statistics, such as $R_{0}$ [2], or to make accurate disease forecasts [3]. In this manuscript, we focus on growth rates rather than $R_{0}$ to reduce the number of modelling assumptions; however, we believe estimates of $R_{0}$ will behave similarly. Overall, there is a trade-off between spatial aggregation, which reduces the random error due to noise, and disaggregation, which reduces bias, much like what is seen in the MAUP and Simpson’s paradox.

## 4. Methods and materials

Data on cumulative COVID-19 infections come from the New York Times [7] as of 31 December 2020. We also collect the population and area within New York City ZCTAs, LA County neighbourhoods, counties and states from the US Census, with population estimates as of July 2019 [17]. States include the District of Columbia. Counties are defined as in the census except for New York City, where all boroughs are combined, and Kansas City, where the population and area are calculated separately. Because Kansas city overlaps with other Missouri counties, we do not remove the city area from our estimates of county areas. We do not expect a significant change in our results due to this decision.

We collected data of US facilities as of 5 May 2020 from the New York Times [5], and infections from New York ZCTAs as of 25 April 2020 from the NYC department of health [18], and from LA County neighbourhoods as of 27 April 2020 from the Los Angeles County Department of Public Health [6]. Data for deaths were not available within LA neighbourhoods. Finally, we collected data across nations from Our World In Data as of 29 April 2020 [8]. Populations and areas of each nation were gathered from the United Nations [19,20].

While there are a number of methods to measure the growth rate of diseases [21,22], two parameter-free methods are commonly used for discrete data, Poisson and negative binomial regression. Because the variance of the growth process does not generally follow a Poisson distribution, we instead fit daily numbers of deaths or infections to the more general negative binomial regression [21]. We define surges in the USA by the following date ranges as we observed that the three surges happened during these periods in most USA regions. The first surge is the exponential growth period that happened before 1 May 2020; the second surge is between 1 May and 1 September 2020; and the third surge is after 1 September 2020. The data grow exponentially in the early phase of surges, as shown in figure 2*a*. To determine the time range in which the disease grows exponentially, we fit the first T datapoints with negative binomial regression, and find the T for which McFadden pseudo-$R^{2}$ is maximized [23]. Figure 2*a* demonstrates that this method can find the appropriate range. For robustness, we removed the cut-off altogether and did not see a change in our qualitative results. We only fit data with more than five datapoints and the total number of infections is greater than 30 in order to reduce statistical noise. From these strict criteria, we fit 116 (out of 223) neighbourhoods in LA. We fit 738 counties with infections and 218 counties with deaths in the USA in the first surge, 1618 counties with infections and 164 counties with deaths in the second surge, 2901 counties with infections and 552 counties with deaths in the third surge. We also fit 50 states in the US plus Washington, DC with infections and 46 states plus Washington, DC with deaths in the first surge, 48 states, plus Washington, DC with infections and 37 states plus Washington, DC with deaths in the second surge, and 50 states plus Washington, DC with infections or deaths in the third surge. In addition, we fit 171 nations with infections and 77 nations with deaths out of 207. In Germany, we fit 390 counties and 16 states with infections, and in Italy we fit 118 provinces and 21 regions with infections.

To demonstrate spatial aggregation bias, we compare the growth rate distributions at different aggregation levels. The significance of differences between two distributions (e.g. county-level and state-level growth rate distributions) are based on the Mann–Whitney U-test, a non-parametric test of the null hypothesis that the median of two distributions are similar. For comparisons between a distribution and the aggregated data (e.g. state-level growth distribution and the aggregated nation-level growth rate), we estimate the error in the distribution mean as the standard deviation divided by the square root of the number of datapoints, and the error in the aggregated growth estimate using standard regression methods. We then propagate errors to check if the mean and aggregate estimate are statistically significantly different.

Last, we specify here how the SIR models used in figure 2*c* were created. We simulated the SIR models based on US county statistics. A total of 100 SIR models were created. The initial numbers of susceptible are taken from the population of 100 randomly drawn US counties, the initial numbers of infected are the number of cases in these corresponding counties 3 days after the first case appeared. The rate from susceptible to infected is drawn from a normal distribution with a mean of 0.14 and a standard deviation of 0.03; and the rate from infected to recovered is drawn from a normal distribution with a mean of 0.02 and a standard deviation of 0.005. These rates were empirically tested to create a various range of disease spreading rates. Finally, we simulated 100 days in each model. The estimated growth of these simulated SIR models shows aggregation bias again, which is consistent with our finding in the empirical data. We then measure the exponential growth rates using negative binomial regression, as before.

## Figures and Tables

**Figure 4.**
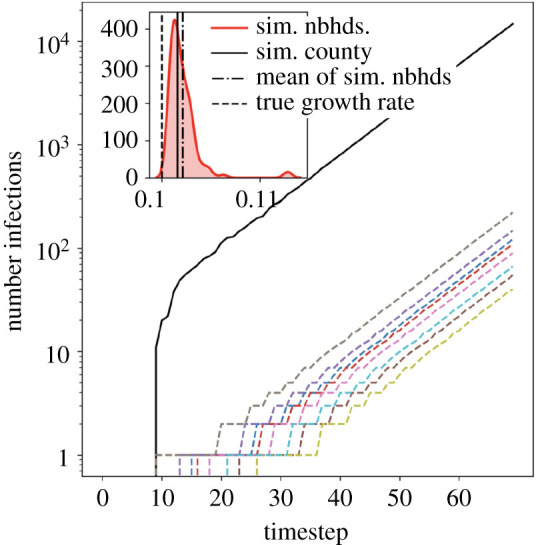
Simulations with the same growth rate but randomized arrival times whose distribution matches that of LA County. (Online version in colour.)

**Figure 5.**
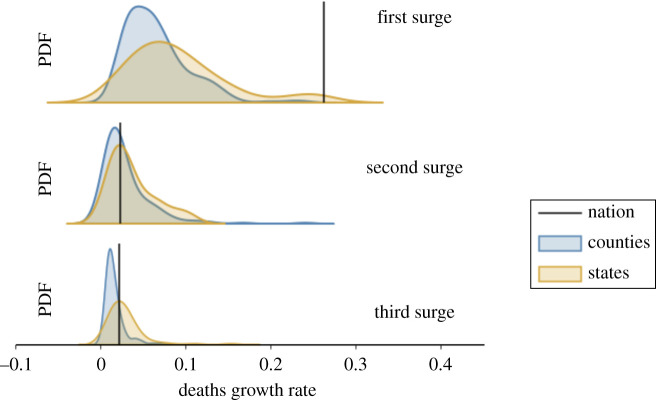
Deaths growth rate distribution for counties, states and the nation for the first, second and third surge. (Online version in colour.)

## Data accessibility

Code is available at https://github.com/fionasguo/covidagg. All data are available from the NYT COVID-19 database (https://github.com/nytimes/covid-19-data), the NYC Health Department (https://github.com/nychealth/coronavirus-data), the Los Angeles Department of Public Health (www.publichealth.lacounty.gov/media/Coronavirus/locations.htm), Our World in Data (https://github.com/owid/covid-19-data), the United Nations (https://population.un.org/wpp/Download/Standard/Population/ and http://data.un.org/Data.aspx?d=FAO&f=itemCode%3A6601), the US Census (https://data.census.gov/cedsci/), the Federal Statistical Office of Germany (https://de.statista.com/statistik/daten/studie/1242/umfrage/bevoelkerungsdichte-in-deutschland-nach-bundeslaendern/), City Population (www.citypopulation.de/en/germany/), National Platform for Geographic Data (https://npgeo-corona-npgeo-de.hub.arcgis.com/) and Population DEMOISTAT (http://demo.istat.it/).

## Appendix A

In this appendix, we add onto our results in the main text through further simulations and empirical data analysis. First, in figure 4, we show simulations in which we keep the growth rate of the simulation fixed for all neighbourhoods (growth rate=0.1), but we match the arrival times of neighbourhoods in LA County. In the inset, we see that, due to finite size effects, the simulated neighbourhoods have growth rates that are slightly above the true value (which are calculated via negative binomial regression). Notice the scale on the x-axis of the inset, which demonstrates that this difference is exceedingly small (less than 5% away from the true result for almost all neighbourhoods). Nonetheless, aggregation produces a subtle, yet statistically significant, difference in the mean growth rate (p-value=0.001).

Next, in figure 5, we show the distribution of deaths growth rates for counties, states and the nation during the first, second and third surges (compare to figure 3 in the main text). We see statistically significant differences in the growth rates with various aggregations at each surge. Namely, we find a significant difference between growth rates at county and state levels (Mann–Whitney U-test $<10^{-14}$ for each surge), and a significant difference between growth rates at the mean state and national level (difference in mean p-value=0.0009). Furthermore, we see statistically significant drops in the infection growth rate at each surge, and at each aggregation level. Namely, the growth rate distributions between the first and second or second and third surges are statistically significantly different at the county level and at the state level (Mann–Whitney U-test $p\text{-value}<10^{-10}$ and <0.01, respectively). The national growth rate also has a significant drop from the first to second and second to third surges (difference in mean $p\text{-values}<10^{-18}$). As in the main text, these growth rates correlate with population density. The Spearman rank correlation for the first surge is 0.28 and 0.62 ($p\text{-value}<10^{-4}$) for counties and states, respectively. For the second surge, the correlations are 0.20 and 0.36 (p-value<0.03) for counties and states, respectively. Finally, for the third surge, the correlations are 0.26 (p-value<10−9) for counties and not significant (p-value=0.66) for states. At the world level, the correlation between nation population density and growth rate is not significant (p-value=0.77). Unlike the correlation between arrival times and infection growth rates, the Spearman rank correlation with arrival times and deaths growth rates for counties and states in the USA are not statistically significant (p-values>0.1). At the world level, the correlation between arrival times in nations and growth rates in nations is significant (−0.29, p-value=0.004). This further confirms the idea that surges are higher early on, when there were fewer effective mitigation strategies.

Last, we show that different aggregation methods can be applied to calculate growth rates in a region. In the main text, we aggregate the daily number of infections or deaths in a region, and fit the exponential curve with negative binomial to estimate the growth rate. The distributions of growth rates exhibit aggregation bias (figures 2 and 3). We can also show these results with an alternative method: we calculate the mean of exponential growth rates of all sub-regions weighted by the number of cumulative infections at the end of the exponential growth phase in the sub-regions. Figure 6 demonstrates that the aggregation bias phenomenon still exists when the alternative aggregation method is applied.
